# Application of CTC-derived spheroid for drug screening toward personalized treatment in patients with breast cancer

**DOI:** 10.1016/j.tranon.2025.102573

**Published:** 2025-10-27

**Authors:** Hsu-Huan Chou, Ting-Fang Che, Kuan-Ju Lee, Shin-Cheh Chen, Jia-Yang Chen, Yen-Jang Huang, Syer Choon Lim, Shih-Chiang Huang, Chia-Lung Tsai, Ying-Chih Chang, Chi-Neu Tsai

**Affiliations:** a Department of General Surgery and Breast surgery, Chang Gung Memorial Hospital Linkou branch, Taoyuan 33305, Taiwan; b Graduate Institute of Clinical Medical Sciences, Chang Gung University, Taoyuan 33302, Taiwan; c Genomics Research Center, Academia Sinica, Taipei 11520, Taiwan; d AcroＣyte Therapeutics Inc., New Taipei City 22175, Taiwan; e Department of Anatomic Pathology, Chang Gung Memorial Hospital Linkou branch, Chang Gung University, College of Medicine, Taoyuan City 33302, Taiwan; f Genomic Medicine Core Laboratory, Chang Gung Memorial Hospital, Taoyuan 33302, Taiwan; g Department of Chemical Engineering, Standford University, Standford, CA 94305, USA; h Developmental Biology and Regenerative Medicine Center, National Taiwan University, Taipei 10672, Taiwan; i Department of Surgery, New Taipei Municipal Tucheng Hospital, (Built and operated by Chang-Gung Medical Foundation), New Taipei City 23652, Taiwan

**Keywords:** Breast cancer, Circulating tumor cells, CTCs spheroid culture, Drug screening, Spatial transcriptomics

## Abstract

**Purpose:**

Tumor heterogeneity and drug resistance remain key challenges in breast cancer management. While organoid cultures can be established from resected tumor specimens, patients undergoing systematic treatment often lack accessible tissue for *ex vivo* drug testing. Circulating tumor cells (CTCs) offer a minimally invasive alternative. The purpose of this study was to establish a clinically feasible workflow that integrates CTC enumeration and CTC-derived spheroid drug screening to overcome the limitations of tissue-dependent organoid models and enable personalized treatment in breast cancer.

**Methods:**

CTCs were isolated from 34 newly diagnosed breast cancer patients using a liposome-tethered supported lipid bilayer (LIPO-SLB) microfluidic platform functionalized with anti-EpCAM antibodies. Thirteen patients undergoing systemic therapy and relapse were further assessed using CTC-spheroid culture for *ex vivo* drug screening. Hormone receptor expression and genomic mutation profiles were integrated with drug testing. One relapsed case underwent pre- and post-treatment spatial transcriptomic analysis (Xenium *in situ*).

**Results:**

CTC and CTC-cluster counts significantly declined post-treatment in responders (*p*=0.044 and *p*=0.0264, respectively), but not in non-responders. CTC-spheroids were successfully generated in all 13 cases, enabling *ex vivo* drug testing. Effective therapies were identified in 9 patients (69.2%), of whom 7 achieved partial responses and 1 achieved stable disease; one patient did not receive the suggested treatment. *Ex vivo* drug sensitivity results correlated with clinical outcomes. Integrating CTC-based drug testing, hormone receptor expression in CTCs, and genomic DNA mutation profile further improved prediction of radiological response. Spatial transcriptomics revealed therapy-induced clonal shifts, with resistant subpopulations characterized by over-expression of chemoresistant gene, EIF4EBP1.

**Conclusion:**

This study demonstrates that CTC-derived spheroid drug screening provides actionable therapeutic guidance when tissue is unavailable. Combined with hormone receptor and genomic profiling, this liquid biopsy–based platform enables personalized treatment, and dynamic monitoring of resistance in breast cancer.

## Highlights

• CTC-derived spheroid drug screening guided therapy in relapsed breast cancer patients.
• *Ex vivo* drug responses closely matched clinical outcomes in tested cases.
• Longitudinal CTC profiling enabled dynamic monitoring of treatment resistance.
• Integration of CTC-based assays improved personalized therapy selection.
• Spatial transcriptomics revealed resistant clones, informing adaptive treatment.

## Introduction

Breast cancer remains the leading cause of cancer-related deaths among women worldwide, with high mortality rates despite advances in treatment over the past decade [1]. Current therapies for advanced breast cancer, including hormone therapy, chemotherapy, targeted therapy, immune checkpoint inhibitor, or combination are generally selected based on standardized guidelines and tailored to the cancer's molecular subtype, genetic mutations, and resistance mechanisms [2,3]. Although treatment guidelines exist for different breast cancer subtypes, selecting an optimal combination of drugs for adjuvant therapy remains a significant challenge, particularly for patients who underwent treatment or have experienced treatment relapses. The heterogeneity of breast tumors, gene expression profiles, tumor mutation burden, and tumor microenvironment contribute to the requirement for refractory treatment [[4], [5], [6], [7]].

Numbers of circulating tumor cells (CTCs) have shown promise as prognostic markers in early or metastatic breast cancer [8]. Detected using the FDA-approved CellSearch System (Janssen), CTCs are identified based on epithelial cell marker epithelial cell adhesion molecule (EpCAM) and are predictive of progression-free, overall survival (OS) and metastatic breast cancer [[9], [10], [11], [12]]. Isolated CTCs can be categorized as pan-cytokeratin (pan-CK)+/CD45-/DAPI+ cells [13]. Additionally, CTCs clusters with white blood cells (WBCs) indicates poorer survival outcomes [14,15]. Most previous studies, investigating CTCs have focused on their prognostic and predictive effects by defining the counts of single cell and clusters of CTCs [16].

In parallel, patient-derived organoids (PDOs) have emerged as powerful *ex vivo* models for cancer research that derived from surgical or biopsy specimens and retain the histological, genetic, and phenotypic features of their parental tumors. Several groups have demonstrated that PDOs from breast cancer faithfully recapitulate tumor heterogeneity and predict treatment response *in vitro*, providing a translational platform for drug testing and precision oncology [17,18]. However, organoid establishment depends on tissue availability, which limits their application in patients undergoing systematic therapy or in relapsed settings where fresh surgical material is lacking.

Besides organoids, emerging research has demonstrated that CTC-derived spheroids can be cultured and used for drug screening across various cancers [[19], [20], [21], [22], [23], [24], [25], [26]]. However, most drugs screened in CTC-spheroids for the abovementioned types of cancer were applied in a limited number of cases or continuous CTC cell lines (**Table S1**) [[19], [20], [21], [22], [23], [24], [25], [26], [27], [28], [29], [30]]. Our previous report demonstrated drug testing using CTC-derived spheroids in a patient with breast cancer [20]. This study builds on our previous findings by expanding drug screening in CTC-spheroids to more patients with breast cancer and integrating genomic and spatial profiling to track resistance. In this study, we evaluated a clinically actionable workflow that integrates CTC enumeration, CTC-derived spheroid drug sensitivity testing to guide therapy and monitor resistance. The potential clinical utility of this platform was also discussed.

## Patients and methods

### Patients

**Fig. 1:**
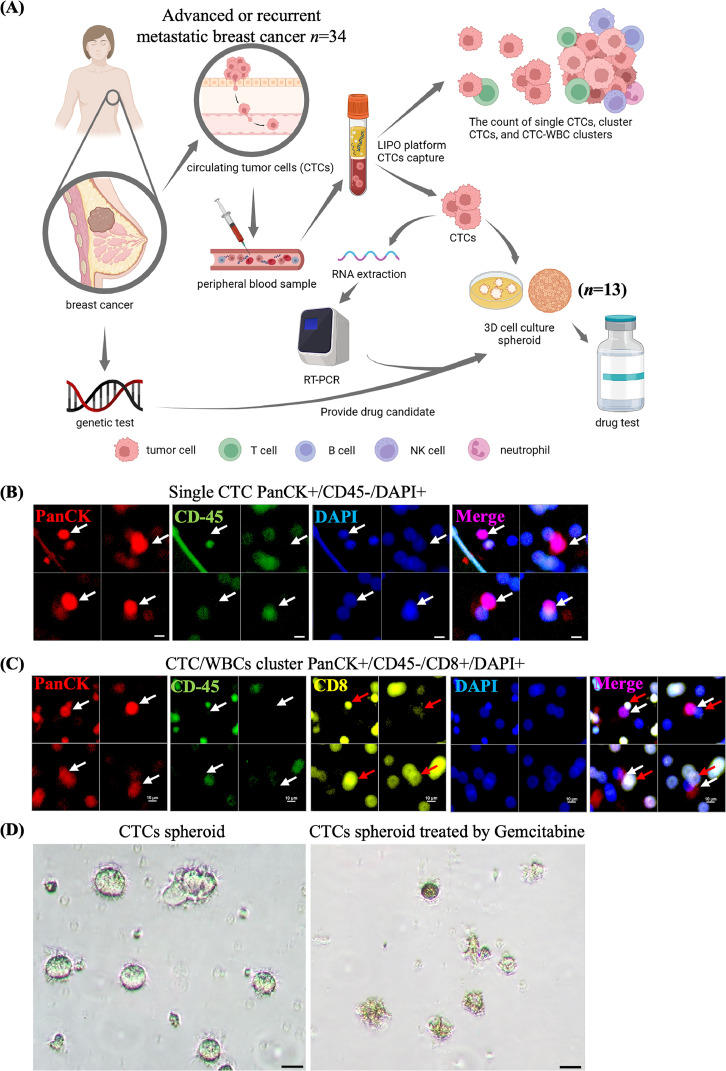
The experimental design of this study and the representative immunofluorescence staining of CTCs, CTC/WBCs cluster, *versus* morphology of CTCs spheroids culture (A) Clinical application of CTCs in advanced or recurrent breast cancer patients. The CTC–white blood cell (WBC) clusters, and CTC-spheroids were isolated from the peripheral blood of breast cancer patients using the LIPO-SLB isolation platform. This schematic was generated with Biorender.com. Both CTC and CTC–WBC counts were counted and correlated with patient prognosis. Additionally, CTCs can be cultured into spheroids for drug screening applications. The integration of genetic testing, clinic-pathological markers, and spatial transcriptomics further enhances the precision of drug selection, leading to more tailored treatment strategies (B)(C). The representative immunofluorescence staining of single CTC (B), CTC–WBC clusters (C) were identified after merging with PanCK, CD45, and DAP. Staining was performed using specific antibodies targeting CD3, CD8, CD4, CD56, and myeloperoxidase (MPO) to distinguish CTC-associated immune cells as T cells, cytotoxic T cells, helper T cells, NK cells, and neutrophils, respectively, in multiple blood samples. (D) The representative cell images of cultured CTC-spheroid without any drug therapy (left panel) The cell images of representative cultured CTC-spheroid after *ex-vivo* drug treatment for 6 days with gemcitabine (right panel). Scale bar =50 μm.

This prospective study enrolled 34 women newly diagnosed with primary locally advanced breast cancer between 2019 and 2022 (**Table S2**). Blood samples were collected from the patients before, during, and after treatment. The patient outcomes were evaluated for a duration of up to 3 years (Fig. 1A). The drug response was determined according to the revised RECIST guidelines (version 1.1) [31]. This study was approved by the Institutional Review Board (IRB) of the Chang Gung Memorial Hospital (IRB number: 202102527B0).

### Immunohistochemistry (IHC) staining

The resection specimens were fixed in formalin, embedded in paraffin (FFPE), sectioned to 4-μm thickness, deparaffinized in xylene, and rehydrated through graded ethanol. Antigen retrieval was performed in citrate buffer (pH 6.0) for 15 min, followed by quenching of endogenous peroxidase with 3% hydrogen peroxide. After serum blocking, sections were incubated with primary antibodies against Estrogen receptor (ER) (M7047; Dako); Progesterone Receptor (PR) (M3569; Dako); and HER2 (AO485 antibody; Dako) for 1 hour at room temperature. Slides were then treated with HRP-conjugated secondary antibodies, visualized with 3, 3'-diaminobenzidine (DAB), counterstained with hematoxylin, dehydrated, and mounted for microscopic evaluation.

### Isolation and characterization of CTCs, CTC-WBCs

The isolation of CTCs and the antibodies used for characterization of CTCs, CTC-WBCs were as previously described [20]. A Nikon Eclipse Ti inverted microscope (Olympus IX-41; 10× magnification) was used to capture fluorescent images.

### RNA extraction and quantitative reverse transcription-polymerase chain reaction (qRT-PCR) analysis

TRIzol reagent solutions (Applied Biosystems, *CA*, USA) were used in accordance with the manufacturer protocol to isolate RNA from CTCs. The reverse transcription reaction was applied by a High-Capacity cDNA Reverse Transcription Kit (Thermo Fisher Scientific) in accordance with the manufacturer protocol. Subsequently, Fast SYBR^TM^ Green Master Mix using a StepOnePlus™ Real-Time PCR System (Thermo Fisher Scientific) was used in accordance with the manufacturer protocol to perform qPCR. The relative gene expression calculated as ΔCT and ΔΔCT, whereas primer sequences, and PCR product size were listed in **Table S3**.

### Three-dimensional (3D) CTC spheroids culture and drug screening

CTC-spheroids were cultured as previously described [20]. Spheroid morphology derived from patient CTCs was observed, and these spheroids were subsequently used for drug screening assays [20]. Staurosporine (STS) served as a positive control for inducing cellular apoptosis. Cell viability was measured using the RealTime-Glo™ Cell Viability Assay (Promega), following the manufacturer protocol. Drug effectiveness was assessed with a response defined as a cell viability below 30% of the control group.

### Xenium *in situ* platform [32]

FFPE tissue samples collected from pre- and post-chemotherapy of patient #8 were processed into Xenium *in situ* slides (Xenium Human Breast Gene Expression Panel, 10X genomics), following protocols described in a previous study [32]. The resulting spatial transcriptomics data were processed with Partek™ Flow™ bioinformatics software (Illumina) and Xenium explorer (10X genomics, USA).

### Statistical analysis

All statistical analyses were performed using IBM SPSS Statistics 21 (IBM Corporation, Armonk, NY, USA). Student’s *t*-test was used to analyze quantitative variables. Statistical significance was set at *p* <0.05.

## Results

### Association between CTC and CTC-WBC counts and clinical outcomes in patients with breast cancer

The demographic characteristics of the 34 patients with breast cancer enrolled in this study are summarized in **Table S2**. Among them, 14 patients had non-metastatic stage II or III disease, while 20 had metastatic tumors. The mean age of the patients was 52 years. Regarding molecular subtypes, 14 patients (41.2 %) had hormone receptor (HR)-positive/HER2-negative tumors, eight (23.5 %) had HR-positive/HER2-positive tumors, four (11.8 %) had HER2-enriched tumors, and eight (23.5 %) had triple-negative breast cancer (TNBC).

As illustrated in Fig. 1A, CTCs (n=34) and CTC–WBC clusters were isolated from peripheral blood using the LIPO-SLB platform with anti-EpCAM antibodies. CTC and CTC–WBC counts were then quantified and analyzed in relation to clinical outcomes. The overall isolation success rate was 94.1 % (32/34). Single CTCs were defined as PanCK⁺/CD45⁻/DAPI⁺ based on merged fluorescence channel images (Fig. 1B). To characterize immune cell components within CTC–WBC clusters, additional staining was performed with antibodies targeting CD3, CD4, CD8, CD56, and myeloperoxidase (MPO) (Fig. 1C). Moreover, CTC spheroid cultures were established from patients undergoing systemic therapy and experiencing relapse, their morphology is shown in Fig. 1D (left panel). These spheroids were treated with various chemotherapeutic agents. Notably, after 6 days of gemcitabine treatment, the spheroids showed marked shrinkage and disrupted morphology (Fig. 1D, right panel), and viability was assessed using a cell viability assay.

Among the 32 patients with successful CTC isolation, the average single CTC count per 2 mL of blood was 31.90 in non-metastatic cases and 85.89 in metastatic cases (*p*=0.4399). The average CTC cluster count was 12.56 and 31.88, respectively (*p*=0.3987), and the total CTC count (single + clustered) was 44.46 in non-metastatic and 117.77 in metastatic patients (*p*=0.4280) (**Supplementary Fig. S1A–S1C**). Among these 32 patients, 26 had paired CTC and CTC cluster counts before and after chemotherapy. Of the 26 patients with longitudinal data, 19 were classified as responders to therapy, while seven were non-responders. In responders, a significant reduction in CTC counts was observed after treatment, including decreases in single CTC (*p*=0.0438), CTC–WBCs (*p*=0.0264), and total CTCs (*p*=0.0191) (**Supplementary Fig. S1D–S1F**). Furthermore, the numbers of CTC-associated CD45⁺ WBCs (*p*=0.0339) and CD3⁺ WBCs (*p*=0.0191) also declined in responders (**Supplementary Fig. S1G–S1H**). In contrast, non-responders exhibited no significant changes in total CTC counts following treatment (**Supplementary Fig. S1I**).

Overall, these findings suggest that a reduction in CTC and CTC–WBC counts after therapy is associated with a favorable treatment response, while persistently elevated counts may reflect resistance to treatment.

### Application of CTC-spheroids for testing clinically used drugs

The *in vitro* CTCs spheroids were established from 13 patients who relapsed from prior treatment or undergoing systemic therapy. The demographic data of these 13 patients were listed in **Table S4**. Initially, commonly used chemotherapy regimens—including anthracycline-, taxane-, and platinum-based treatments, as well as their combinations—were tested to assess their effects on CTC-spheroid viability *in vitro*. Drug sensitivity screening identified effective treatments for nine cases (69.2 %) of 13 patients. Among them, seven responded favorably, one exhibited stable disease, and one did not receive the matched drug. However, four patients of 13 patients could not find any sensitive drugs using CTC-spheroid platform, the outcome of these four patients varied.

**Fig. 2:**
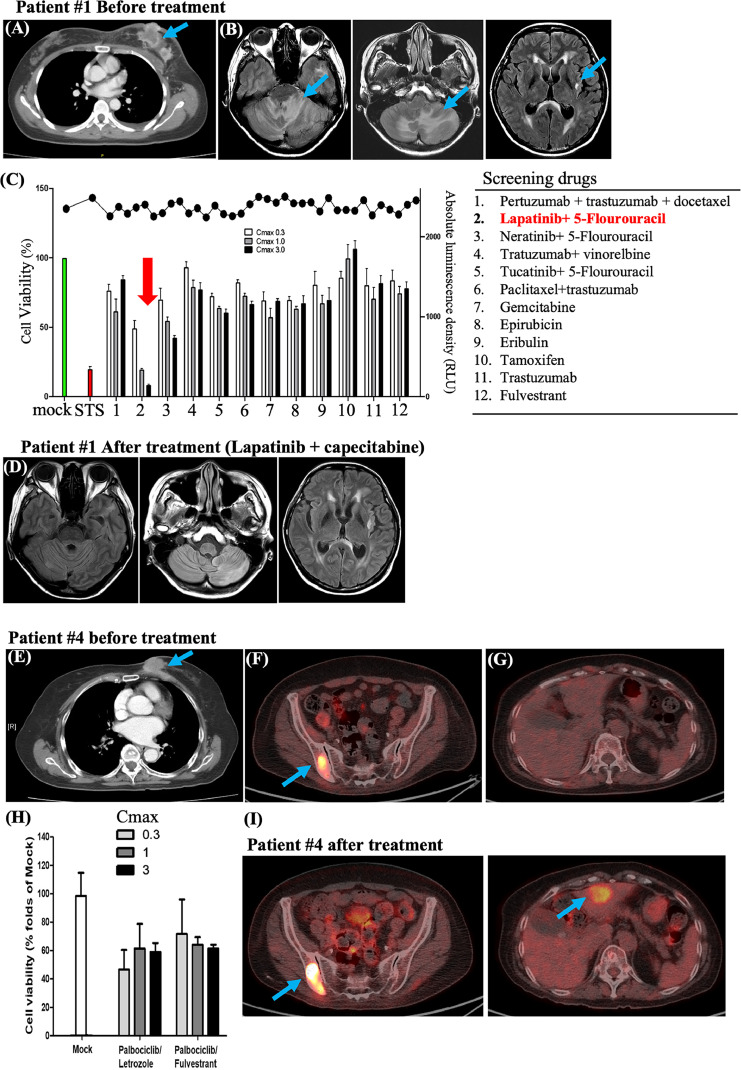
**CTC-spheroid drug screening outcomes and corresponding patient clinical responses** (A) Patient 1 was a woman diagnosed with left breast cancer (B) who initially presented with brain metastasis. (C). The CTC-spheroid culture of patient #1 were applied for drug screening, the screening drugs was as right panel listed. The arrowhead indicated the isolated CTCs used for drug screening revealed sensitivity to lapatinib and 5-FU. (D). Partial response of brain metastasis was observed after treatment of lapatinib and capecitabine for 6 months in patient #1. (E) Patient 4 was a woman with left breast cancer presenting with multiple metastasis, (F) the formation of new bony metastasis was observed, (G). Without liver metastasis (H) Drug test conducted at the time of first progression revealed resistance to palbociclib + letrozole and palbociclib + fulvestrant. (I) Standard second-line therapy using a combination of fulvestrant and palbociclib was commenced. Disease progression occurred after 3 months in patient #4, with the bone and liver metastasis progressing rapidly.

Patient 1 was a 46-year-old woman with HR-positive HER2-positive breast cancer with bone and brain metastases. Docetaxel combined with dual blockade (trastuzumab and pertuzumab) was used as the first-line palliative treatment. A partial clinical response was observed in the breast tumor (Fig. 2A, arrowhead); however, brain metastasis continued to progress (Fig. 2B, arrowhead), and the CTCs were isolated for spheroid culture at this point. The isolated CTC-spheroid showed sensitivity to lapatinib + 5-FU (Fig. 2C, red arrowhead). The clinical partial response of the brain metastasis after patient #1 received treatment with combination of lapatinib and capecitabine for 6 months (Fig. 2D). Similarly, Patients 2 and 3 also showed partial responses when treated with drugs identified as effective in CTC-spheroid screening (**supplementary Fig. S2, S3**). These findings suggested that *in vitro* CTC-spheroid drug screening could help guide personalized treatment by identifying more effective therapies.

Patient 4 was an 82-year-old woman with left HR-positive HER2-negative breast cancer who presented with bone and liver metastases. The patient was undergoing first-line treatment with letrozole + palbociclib and exhibited good disease control before treatment and adequate response (Fig. 2E), however local disease progression and new bone metastases were also observed (Fig. 2F and 2G). Drug tests on patient’s CTCs spheroid conducted at the time revealed resistance to palbociclib + letrozole or palbociclib + fulvestrant (Fig. 2H). Patients received the standard second-line treatment with fulvestrant + palbociclib, the disease progressed after 3 months, and bone metastasis progressed rapidly (Fig. 2I).

These case studies demonstrate that CTC-spheroid-based drug screening can provide valuable insights into treatment selection. The strong correlation between *in vitro* CTC-spheroid drug sensitivity results and actual clinical outcomes.

### Longitudinal application of hormone receptor expression in CTCs and CTC-Spheroid drug screening

**Fig. 3:**
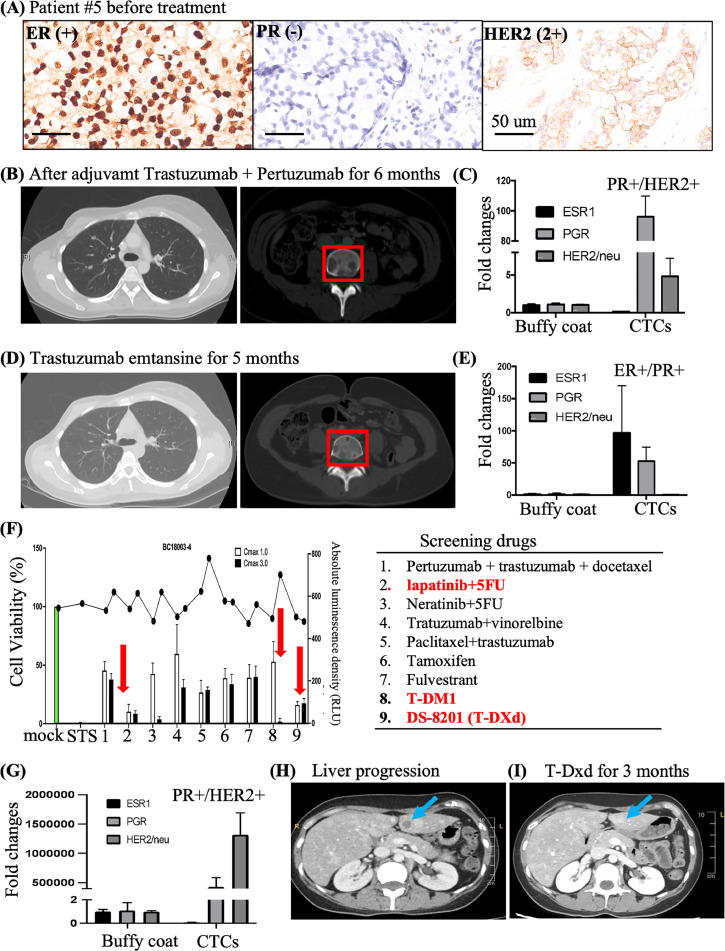
**Application of expression of hormone receptor in CTCs for spheroid drug screening** (A) Patient 5 was a woman with right locally advanced, ER-positive, PR-negative breast cancer with HER2 overexpression. (B) Lung and bone metastasis developed 6 months after the completion of adjuvant treatment. (C) RNA extraction and RT-PCR analysis from the isolated CTCs after treatment revealed higher expression levels of PR and HER2. (D) Partial response in lung and bone metastasis was observed after anti-HER2 treatment as T-DM1 for 5 months. (E) RNA expression analysis performed again using CTCs revealed the level of HER2 as negative control after T-DM1 treatment. (F) The CTC spheroids isolated for drug test revealed sensitivity to lapatinib combined with 5-FU and trastuzumab deruxtecan (T-DXd). T-DM1 exhibited lower inhibition of spheroid. (G) RNA expression in the CTCs revealed higher expression of HER2 and PR after the discontinuation of T-DM1. (H) The liver metastasis progressed, (I) Good response to T-DXd for liver metastasis.

Patient 5 was a 30-year-old woman who presented with right locally advanced, estrogen receptor (ER)-positive, progesterone receptor (PR)-negative, HER2-positive breast cancer undergoing neoadjuvant treatment and nipple-sparing mastectomy with implant reconstruction (Fig. 3A). Lung and bone metastases were detected 6 months after the completion of adjuvant treatment (Fig. 3B). The isolated CTCs and the WBC buffy coat were collected after treatment for RNA extraction and RT-PCR analysis. The WBC buffy coat was used as the negative control. The expression of ER was as low as that of the negative control after 6 months of Trastuzumab + Pertuzumab treatment (Fig. 3C). Based on the result, this patient received an anti-HER2 treatment with T-DM1, lung and bone metastases exhibited partial response to treatment (Fig. 3D). After 5 months T-DM1 treatment, RNA expression elevated levels of ER and PR, the expression of HER2 was like that observed in the negative control (Fig. 3E). At this point, CTC spheroids were applied for drug testing revealed sensitivity to lapatinib + 5-FU + T-DXd, T-DM1 (Fig. 3F). However, hormone treatment with tamoxifen + a GnRH analog was commenced for economic reasons of this patient, resulting in the discontinuation of T-DM1. After 2 years of stable disease, patient received T-DM1 treatment again owing to RNA expression in the CTCs, indicating higher expression of HER2 and PR (Fig. 3G). In addition, progression of liver metastasis was observed (Fig. 3H, arrowhead), therefore the patient subsequently received T-DXd therapy exhibiting good disease control which was compatible with the result of CTCs spheroids drug test (Fig. 3I, arrowhead). Over a period of more than five years, the patient was monitored longitudinally, with hormone receptor expression in CTCs and CTC-spheroid drug screening platform optimized treatment selection.

In summary, drug sensitivity results from CTC spheroids aligned with clinical responses, demonstrating the utility of integrating CTC profiling and CTC-spheroid testing for personalized therapy in metastatic breast cancer.

### Application of DNA mutation data of tumor lesions for CTC spheroid drug screening

**Fig. 4:**
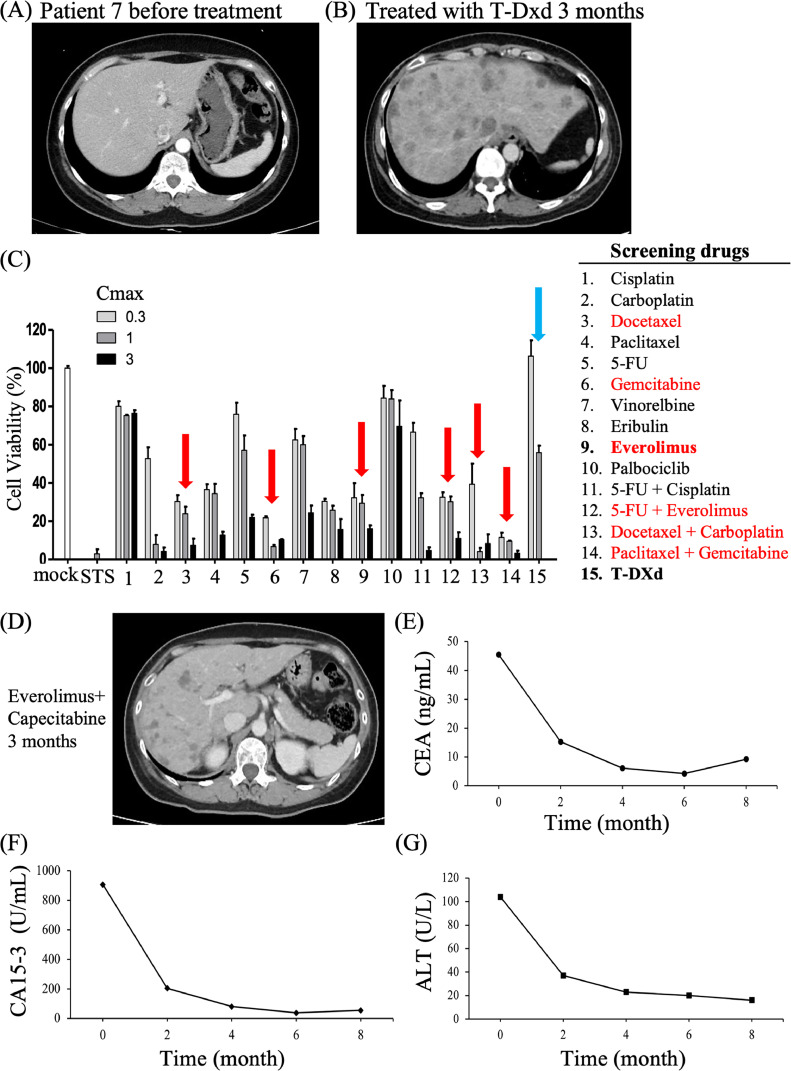
**Integration of Genetic Mutation Results for drug screening in CTC spheroid culture** (A) Patient 7 was woman with recurrent left breast cancer who presented with lung metastasis after receiving multiple lines of hormone therapy and chemotherapy. (B) Liver metastasis developed. (C) Drug screening using CTC spheroids revealed good response to mTOR inhibition and taxane-based chemotherapy. (D) A dramatic response was observed as multiple liver metastases regressed after treatment with a combination of mTOR inhibitor + capecitabine. (E∼G) The tumor marker levels have decreased markedly, and liver function has returned to within the normal range.

Patient 7 was a 56-year-old woman with recurrent left luminal breast cancer who presented with lung and liver metastases after receiving multiple rounds of hormone therapy and chemotherapy (Fig. 4A). A core needle biopsy of the left axillary lymph node revealed metastatic carcinoma with 5 % low-positive ER, negative PR, and HER2 2+ on IHC staining. T-DXd, a monoclonal anti-HER2 antibody drug conjugate, was administered as the HER2 expression was low. However, progression of the liver metastasis was also observed (Fig. 4B). The tumor lesions of the patients were re-examined and revealed a pathological AKT mutation (p.E17K) on the NGS platform (TrueSight Oncology 500). A specific inhibitor targeting mTOR in combination with other chemotherapy drugs was applied for drug screening using CTC-spheroids owing to the presence of AKT gene mutation (Fig. 4C). The drug screening results in CTCs spheroids indicated that mTOR inhibition and taxane-based chemotherapy resulted in a good response (as red arrowhead indicated), whereas the T-DXd had no inhibitory effect on spheroid growth (light blue arrowhead) (Fig. 4C, left panel) as consistent with previous clinical response to T-Dxd of this patient. Patient 7 received the combination of mTOR inhibitor with capecitabine, a dramatic response was observed as the multiple liver metastases regressed after continuing for 2 months (Fig. 4D). The tumor marker levels have decreased markedly, and liver function has returned to within the normal range and stable disease was observed for over 12 months (Fig. 4E to G). Thus, the DNA mutation data in tumor facilitated drug screening in CTC spheroid toward precise medicine.

### Tumor heterogeneity affected therapeutic effects in patients

Patient 8 was a 67-year-old woman with left clinical stage III TNBC (**Fig. S4A**) who received neoadjuvant chemotherapy with docetaxel + cisplatin based on drug screening results from CTC-spheroid culture (**Fig. S4B**). The partial response to chemotherapy was impressive (**Fig. S4C**). However, disease progression was observed after changed drugs with four cycles of anthracycline-based neoadjuvant chemotherapy (**Fig. S4D**). Only one CTC was detected in all blood samples before treatment, whereas an increase in CTCs and CTC-WBCs counts (CD45 or CD3) was observed after anthracycline-based neoadjuvant chemotherapy (**Fig. S4E and S4F**). To explore the reason for treatment relapse, the pre- and post-treatment specimens were analyzed using a spatial transcriptome platform in the following study.

**Fig. 5:**
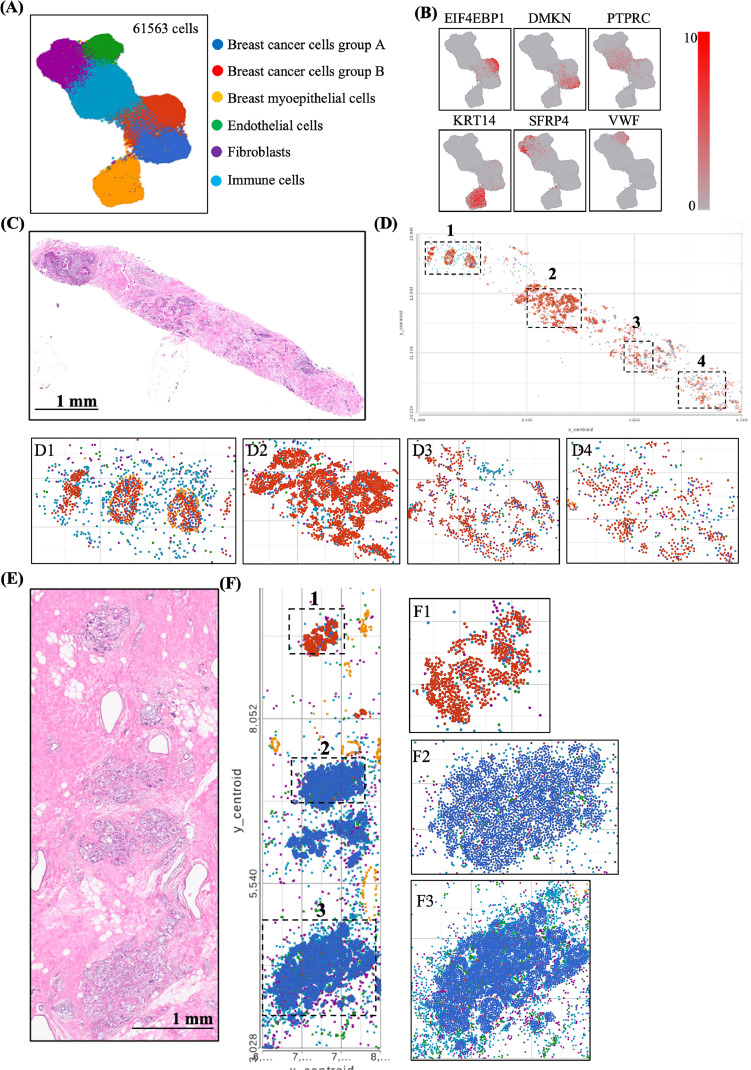
**Analysis of the pre- and post-treatment tissue specimens through Xenium spatial transcriptome** (A) Analysis of the pre- and post-treatment tissue specimens revealed clustering of total single cells into six groups: pre-treatment breast cancer cells, post-treatment breast cancer cells, myoepithelial cells, endothelial cells, fibroblasts, and immune cells. (B) The representative gene expression for each cell type was as revealed. (C) Hematoxylin and eosin (H&E) stain of the pre-treatment specimen. (D) Spatial transcriptome. The distribution of the tumor cells and immune cells was observed in different tumor foci (D1∼D4), showing major tumor subgroup was group B (red color) with few group A tumor (blue color). (E) Hematoxylin and eosin (H&E) stain of the post-treatment specimen. (F) Spatial transcriptome of post-treatment specimen. Group B tumor (red color) was largely eliminated following treatment with taxanes and anthracycline-based regimens. Thus, group A tumor cells (blue color) were dominant after chemotherapy (F1∼F3).

**Fig. 6:**
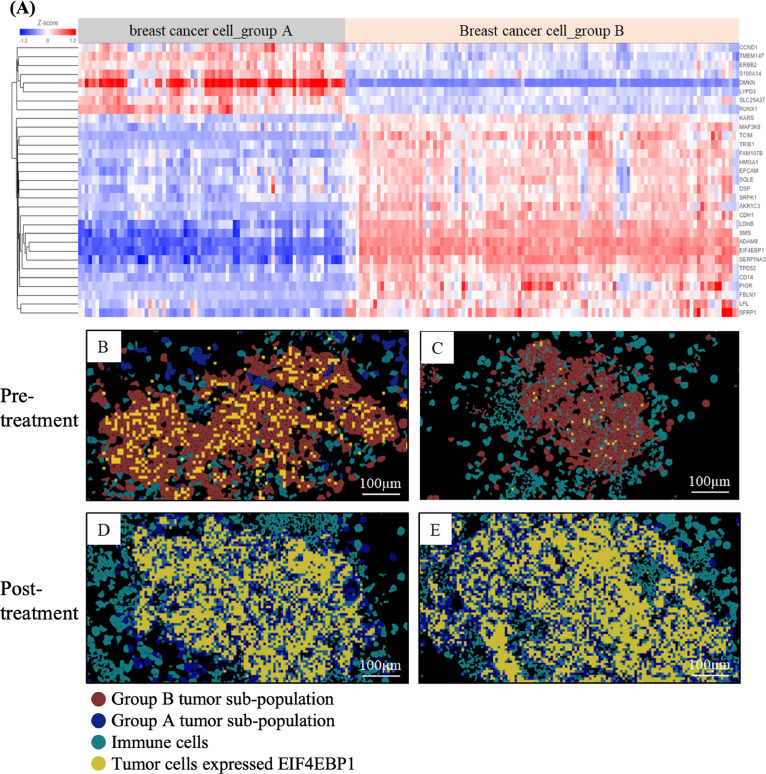
**The gene expression profile in tumor specimen before and after treatment.** (A) Differential gene expression in two types of breast cancer lesions before and after anthracycline-based treatment in Patient #8. Heatmap illustrating differential gene expressions between two distinct tumor subtypes in the same patient. Gene overexpression is shown in red, and downregulation is shown in blue. (B-E) Spatial expression of EIF4EBP in pre- and post-treatment tumor lesions. EIF4EBP expression is shown in yellow, while tumor subgroups A and B are marked in blue and red, respectively.

Analysis of patient samples, including pre- and post-treatment tissue specimens (Fig. 5A), revealed clustering of single cells into six groups: pre-treatment breast cancer cells, post-treatment breast cancer cells, myoepithelial cells, endothelial cells, fibroblasts, and immune cells (Fig. 5A). The representative gene expression for each cell type was as revealed in Fig. 5B. Fig. 5C presented hematoxylin and eosin (H&E) staining of pre-treatment specimen, whereas Fig. 5D presents the tumor cell types analysis using spatial transcriptome. Two tumor subpopulations (Group A: blue, Group B: red) were initially present in pre-treatment lesions (Fig. 5D1∼4), with Group B being predominant. However, Group B was largely eradicated following taxane- and anthracycline-based chemotherapy, allowing Group A to become dominant post-treatment (Fig. 5E and 5F). The dramatic differential gene expression between group A and B cancers was listed in Fig. 6A. The Eukaryotic Translation Initiation Factor 4E Binding Protein 1 (EIF4EBP1), ADAM Metallopeptidase Domain 9 (ADAM9), Serpin Family A Member 3 (SERPINA3), Spermine Synthase (SMS), Lactate Dehydrogenase B (LDHB), Tumor Protein D52 (TPD52), and Secreted Frizzled Related Protein 1 (SFRP1) genes were highly expressed in post-treatment specimen (group A cancer), which are associated cellular metabolism, chemo-resistance or epithelial-mesenchymal transition pathway [[33], [34], [35], [36], [37], [38]]. Dermokine (DMKN), involved in differentiation of epithelial cells [40], was the most downregulated gene in post-treatment specimen (group A cancer). The EIF4ABP1 was highly expressed in post-treatment specimen (group A cancer) as compared with pre-treatment specimen (group B cancer) (Fig. 6B-6D). EIF4BP1 was regulated by the PI3K-mTOR pathway, which plays a crucial role in cell proliferation and chemoresistance [39,40]. This case highlights how intratumoral heterogeneity critically shape treatment outcomes in TNBC. Although initial chemotherapy eliminated the dominant Group B tumor population, a therapy-resistant Group A subclone with enhanced metabolic activity, EMT features, and PI3K–mTOR–EIF4EBP1 signaling emerged, driving relapse. These findings underscore the importance of integrating CTC-based functional assays with spatial transcriptomics to reveal dynamic clonal shifts and resistance mechanisms, thereby guiding adaptive and personalized treatment strategies.

## Discussion

To our knowledge, this is the first study to integrate CTC analysis, CTC-derived spheroid drug screening, and hormone receptor expression, and spatial transcriptomic into a unified workflow for breast cancer management. This approach directly addresses a major barrier to clinical translation: organoid models, while powerful, require surgical or biopsy tissue [17] and are not feasible for patients undergoing systemic therapy. Previous reports demonstrated that breast cancer CTCs can be cultured as continuous cell lines and subsequently applied in drug susceptibility assays (**Table S1**) [19], providing experimental models to investigate metastasis and therapeutic vulnerabilities [41]. Building upon these observations, our study showed that the CTC-derived spheroid culture could provide treatment selection for patients, furthermore the clinical outcome along well with *in vitro* CTC-spheroid drug sensitivity results, which could provide clinician real-time therapeutic suggestions to choose suitable drug(s) before treatment, thereby providing clinicians with a tissue-independent solution. This highlights the potential of CTC-based platforms to offer real-time therapeutic guidance for clinicians when tissue biopsies are limited or unavailable.

Furthermore, RNA expression analysis of CTCs (patient 5, Fig. 3) allowed longitudinal monitoring of hormone receptor changes and disease progression through liquid biopsy. Integration of CTC-spheroid drug screening and hormone receptor changes, patients have survived over five years after diagnosed. In patient 8, the CTC or CTC-WBCs cluster was rare before treatment but increased after therapy (**Fig. S4**). The CTC-WBC cluster in patient #8 showed the PanCK+/CD3+ (**Fig. S4E, S4F**), however the subtype of T cells was not identified in this study. Reviewing literatures, the increasing number of CTC-WBC clusters were the indicator for poor prognosis of patients with cancers [[42], [43], [44], [45], [46]]. Therefore, the cell types/subtype in CTC-WBC cluster should be clarified, which might represent the TME and provided the information about disease progression.

Additionally, compared with existing approaches, our workflow offers distinct advantages. PDOs are valuable *ex vivo* systems that recapitulate tumor biology but require fresh tissue, are labor-intensive, and have variable success rates [17,18]. Continuous CTC lines have been reported but remain rare and not widely generalizable [19]. In contrast, our workflow achieved a 94.1 % CTC capture rate, consistent spheroid formation in all tested patients, and rapid turnaround for drug testing, highlighting its clinical practicality. The ability to perform repeated sampling via liquid biopsy also allows longitudinal monitoring of therapeutic response, which is not feasible with PDOs. Together, these features underscore the advantages of our CTC-spheroid platform as a real-world tool for personalized oncology.

Tumor heterogeneity leads to variations in treatment responses. In patient 8, the group A cancer became dominant after being treated with anthracycline-based neoadjuvant chemotherapy (Fig. 5E, blue color). Based on the gene expression profile, EIF4EBP1 highly expressed in group B cancer (Fig. 6A), which has been reported to contribute for tamoxifen resistance in breast cancer [39]. Anthracycline-based neoadjuvant chemotherapy could induce ferroptosis, lipid peroxidation, and produced excess ROS through drug action [47]. Tamoxifen acts as the antagonist for estrogen to block its binding with estrogen receptor in breast cancer [48]. Nevertheless, since the expression of EIF4EBP1 might indicate the activation of PI3K-mTOR pathway, therefore the downstream expression of autophagy was also dys-regulated [39]. Recent reports showed that autophagy could modulate tumor metastasis and drug resistance in patients with breast cancer [49,50]. Therefore, the link of expression of EIF4EBP1 with resistance to Anthracycline-based neoadjuvant chemotherapy in patients with breast cancer should need further investigation.

In addition to chemotherapy and targeted therapy, our findings have implications for immunotherapy. We observed increases in CTC–WBC clusters, including PanCK⁺/CD3⁺ phenotypes (**Fig. S4**), after treatment in patient #8. Such clusters may represent tumor–immune interactions and could serve as biomarkers of immune evasion. Although immunotherapy agents were not directly tested in this study, integrating CTC profiling with PD-L1 expression, tumor mutational burden, or immune gene signatures may extend this platform to predict response to immune checkpoint inhibitors. Specifically, we are currently establishing a co-culture platform combining patient-derived CTCs and immune cells, which allows real-time assessment of tumor–immune interactions and therapeutic responses. A preliminary illustration has been added (**Fig. S5**) to show this concept: CTCs and immune cells isolated from peripheral blood are co-cultured enabling visualization of immune–tumor cell interactions and subsequent response profiling to immunotherapy. Thus, CTCs analysis not only informs drug sensitivity but also offers a minimally invasive window into the tumor microenvironment, supporting future applications in immunotherapy guidance.

Importantly, our integrated workflow demonstrated high capture efficiency, consistent spheroid growth, and strong clinical correlation, overcoming limitations of prior CTC counting or tumor organoid models. By bridging functional drug testing with molecular profiling, this liquid biopsy–based platform provides a clinically actionable, real-time approach to personalized therapy in breast cancer.

## Conclusion

In this study, we employed CTC-derived spheroid drug screening and hormone receptor expression profiling to guide drug selection prior to clinical administration in patients and closely reflect patient outcomes in breast cancer. Additionally, integrating DNA mutation profiling and RNA expression analysis from CTCs contributed to the development of personalized treatment strategies. Spatial transcriptomics further revealed that tumor heterogeneity significantly influences therapeutic outcomes in breast cancer patients. Together, these findings establish a minimally invasive, clinically actionable workflow that may support personalized treatment decisions and dynamic monitoring of resistance mechanisms in patients with breast cancer.

## Ethics approval and consent to participate

This study was approved by the ethics committee of Chang-Gung Memorial Hospital (institutional review board [IRB] number 202102527B0). The requirement for informed consent was waived by the IRB of the Chang Gung Medical Foundation.

## Trial registration

ISRCTN10116660.

## Funding

This study was supported by the research plan of Chang Gung Memorial Hospital (CMRPG1K0061 and CMRPG1M0141 for Dr. H.H. Chou) and the National Science and Technology Council, Taiwan, under grant numbers 110-2823-8-001-001 and 109-2823-8-001-003 (to Y.C. Chang).

## Availability of data and materials

Data are available from Dr. HH Chou upon request.

## CRediT authorship contribution statement

**Hsu-Huan Chou:** Writing – review & editing, Writing – original draft, Methodology, Investigation, Funding acquisition, Conceptualization. **Ting-Fang Che:** Methodology, Investigation. **Kuan-Ju Lee:** Software, Methodology. **Shin-Cheh Chen:** Resources, Formal analysis. **Jia-Yang Chen:** Methodology. **Yen-Jang Huang:** Methodology. **Syer Choon Lim:** Methodology. **Shih-Chiang Huang:** Validation, Methodology. **Chia-Lung Tsai:** Methodology. **Ying-Chih Chang:** Writing – original draft, Supervision, Conceptualization. **Chi-Neu Tsai:** Writing – review & editing, Writing – original draft, Conceptualization.

## Declaration of competing interest

The authors declare that they have no known competing financial interests or personal relationships that could have appeared to influence the work reported in this paper.

## Appendix Supplementary materials

Image, application 1

Image, application 2
