# Supplementary material for: Application of CTC-derived spheroid for drug screening toward personalized treatment in patients with breast cancer
Source: Transl Oncol. 2025 Oct 27;63:102573. doi: 10.1016/j.tranon.2025.102573 (PMC12597000; doi:10.1016/j.tranon.2025.102573)
Supplement: Supplementary file 1 [file mmc1.docx]

**Application of CTC-Derived Spheroid for Drug Screening toward Personalized Treatment in patients with Breast Cancer**

**Short running title:** CTC-Spheroids for drug screening in patients with Breast Cancer

Hsu-Huan Chou, Ting-Fang Che, Kuan-Ju Lee, Shin-Cheh Chen, Jia-Yang Chen, Yen-Jang Huang, Syer Choon Lim, Shih-Chiang Huang, Chia-Lung Tsai, Ying-Chih Chang#, Chi-Neu Tsai^#^

**Supplementary information**

**Figure S1. Comparison of Circulating Tumor Cell (CTC) Counts Between Non-Metastatic and Metastatic Breast Cancer Patients, and the Dynamic Changes in CTC Clusters Among Clinical Responders & non-responder**

**Figure S2. Clinical response before and after CTC-spheroid drug screening in patient #2**

**Figure S3. Clinical response before and after CTC-spheroid drug screening in patient #3**

**Figure S4.** **Clinical response before and after CTC-spheroid drug screening in patient #8**

**Figure S5. A preliminary illustration demonstrating the co-culture of CTCs and immune cells. for potential application in immunotherapy.**

**Table S1. The CTCs cultures have been applied for drug screening in cell lines or patients with cancers**

**Table S2. Demographic data of enrolled patients in this study**

**Table S3. Primers used in this study**

**Table S4 The patients enrolled for drug test using CTC-spheroid culture**


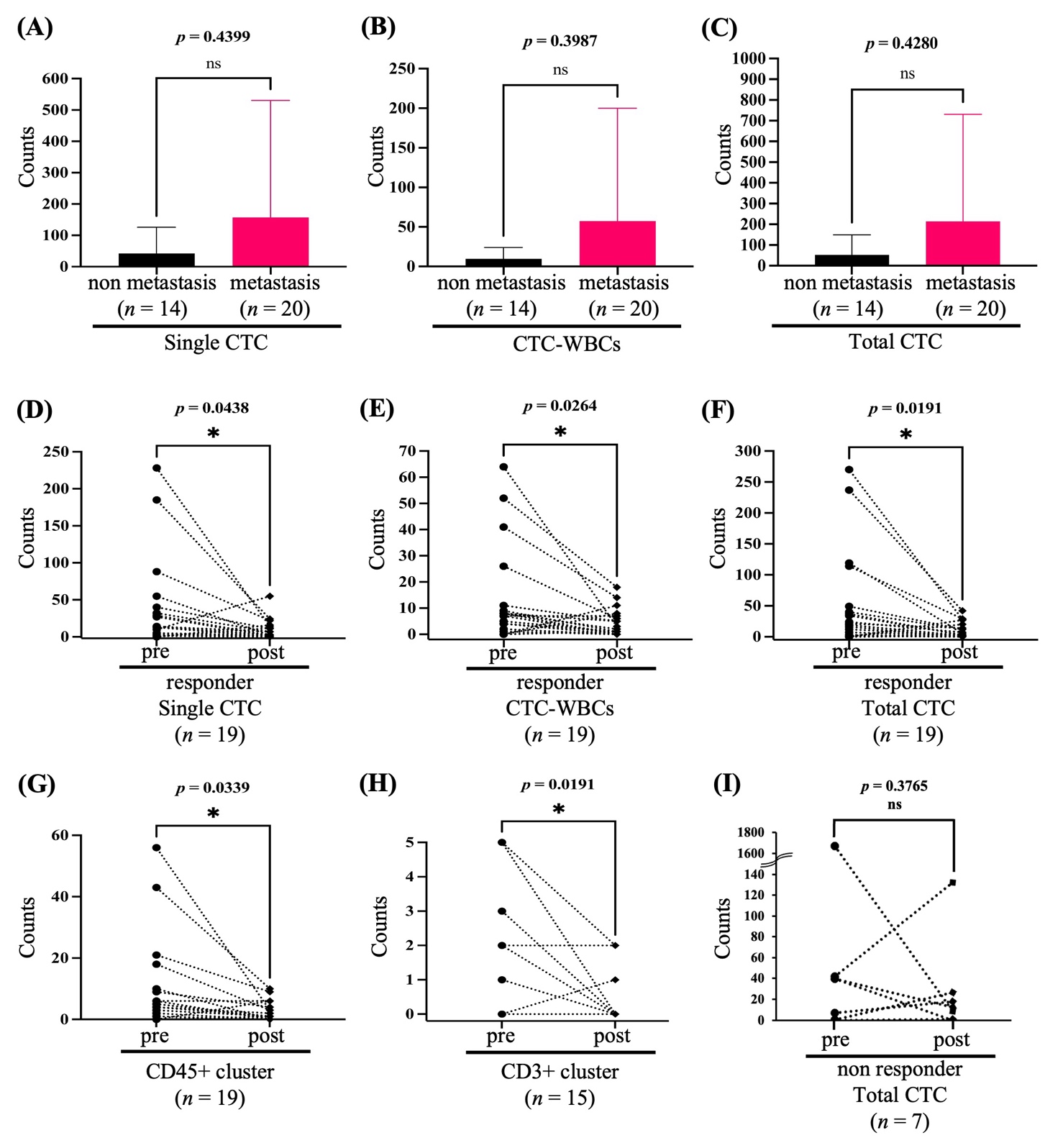


**Figure S1.** Comparison of Circulating Tumor Cell (CTC) Counts Between Non-Metastatic and Metastatic Breast Cancer Patients, and the Dynamic Changes in CTC Clusters Among Clinical Responders & non-responder. (A) The single CTCs counts in patients with non-metastatic and metastatic breast cancer (B) The cluster CTCs counts in patients with non-metastatic and metastatic breast cancer (C) The total CTCs counts in patients with non-metastatic and metastatic breast cancer. The difference between the CTC and CTC-WBC counts before and after treatment in patients with breast cancer. (D) The single CTCs counts. (E) The cluster CTCs counts (F) The total CTCs counts. (G) CD45+ cluster CTCs counts. (H) CD3+ cluster CTCs counts in clinical responder. (I). The total CTCs counts before and after treatment in patients with non-responsiveness. **p*<0.05, Tx: treatment. ns: non-significant.

| **Screening Drugs** |
| --- |
| 1. Cisplatin |
| 2. 5-FU |
| 3. Gemcitabine |
| 4. Vinorelbine |
| **5. Trastuzumab** |
| 6. Eribulin |
| **7. Docetaxel** |
| 8. Carboplatin |
| 9. Paclitaxel |
| 10. Doxorubicin |
| 11. Epirubicin |


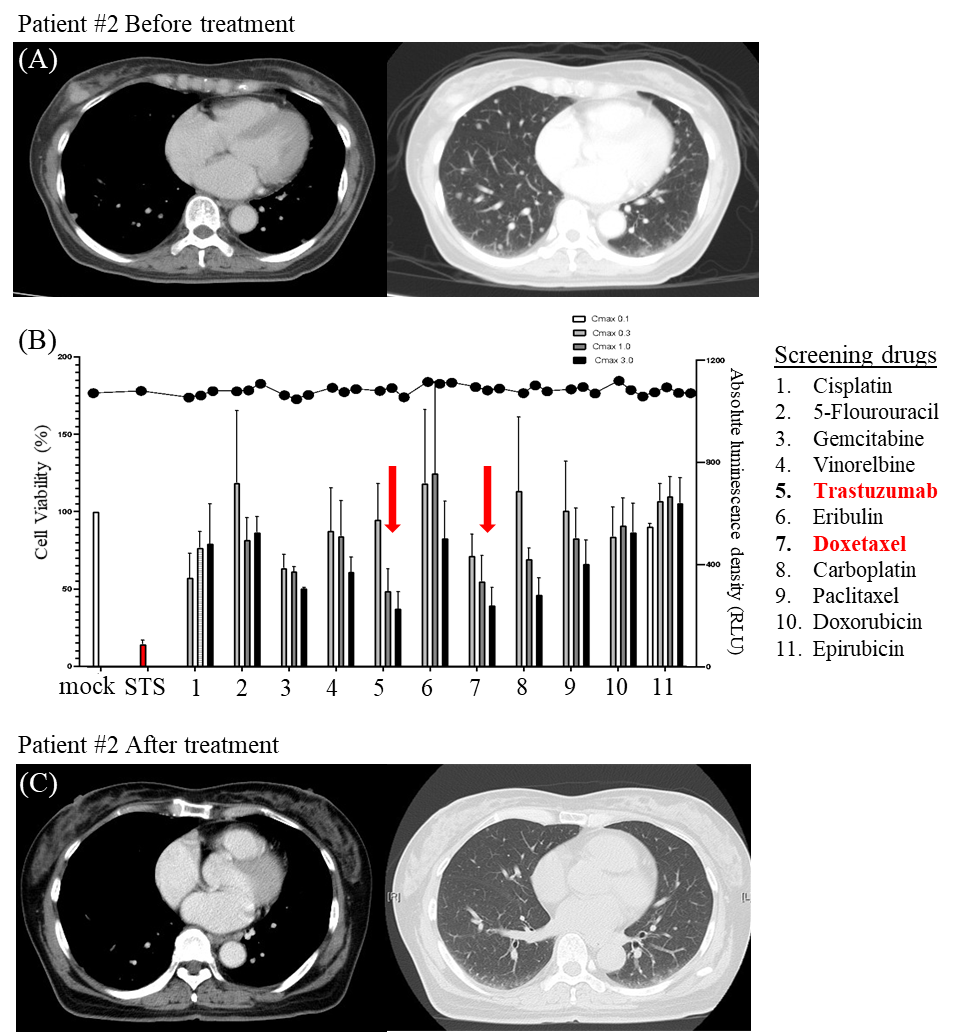


**Figure S2. Clinical response before and after CTC spheroid drug screening in patient #2** (A) Patient 2 was a 52-year-old woman with HR-positive HER2-positive breast cancer with lung metastases after treatment. (B) The drug test performed using CTCs spheroids revealed relative sensitivity to trastuzumab and docetaxel. (C) Good response was observed after trastuzumab and docetaxel treatment.


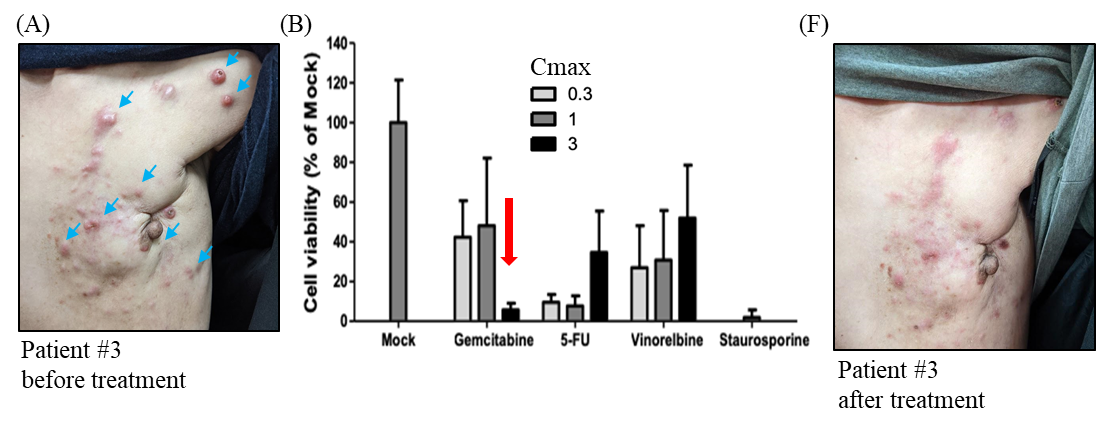


**Figure S3. Clinical response before and after CTC spheroid drug screening in patient #3** (A) Patient 3 was a 51-year-old woman with left breast cancer, HR-negative, HER2-positive breast cancer. Local recurrence at the chest wall occurred after receiving neoadjuvant chemotherapy and trastuzumab-based targeted therapy and undergoing left modified radical mastectomy, nevertheless the TNBC subtype occurred several months after the surgery. Local recurrence progressed to regional skin carcinomatosis after extensive treatment with sequential polychemotherapy combining paclitaxel, eribulin, lipo-doxorubicin, vinorelbine, capecitabine, and cyclophosphamide (B) Drug testing performed at the time of progression revealed sensitivity to gemcitabine. (C) The clinical response of the patient was obvious and the carcinomatosis in the regional skin diminished after treatment with gemcitabine.


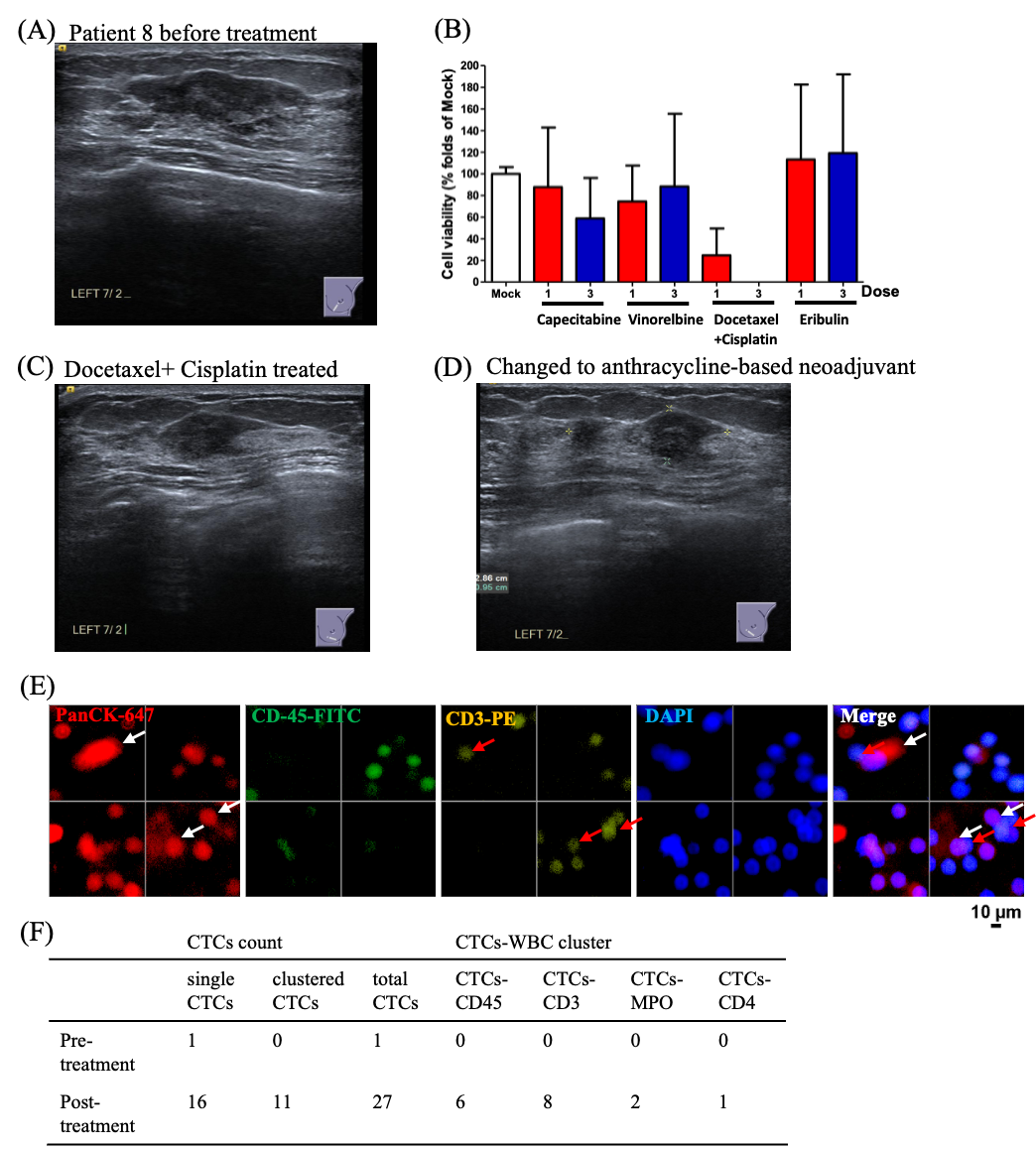


**Figure S4.** **Clinical response before and after CTC spheroid drug screening in patient #8** (A). Patient 8 was a 67-year-old woman with left clinical stage III TNBC. (B) Blood sample obtained before chemotherapy and the result of drug test revealed response to docetaxel + cisplatin and resistance to other drugs. (C) Partial response was impressive after chemotherapy. (D) Disease progression was observed during neoadjuvant chemotherapy with anthracycline-based regimens. (E) Numerous CTCs and CTCs-WBC were detected after anthracycline-based drug treatment by immunostaining. (F) The counts of CTCs, CTC-WBC before and after anthracycline-based regimens treatment in patient #8.


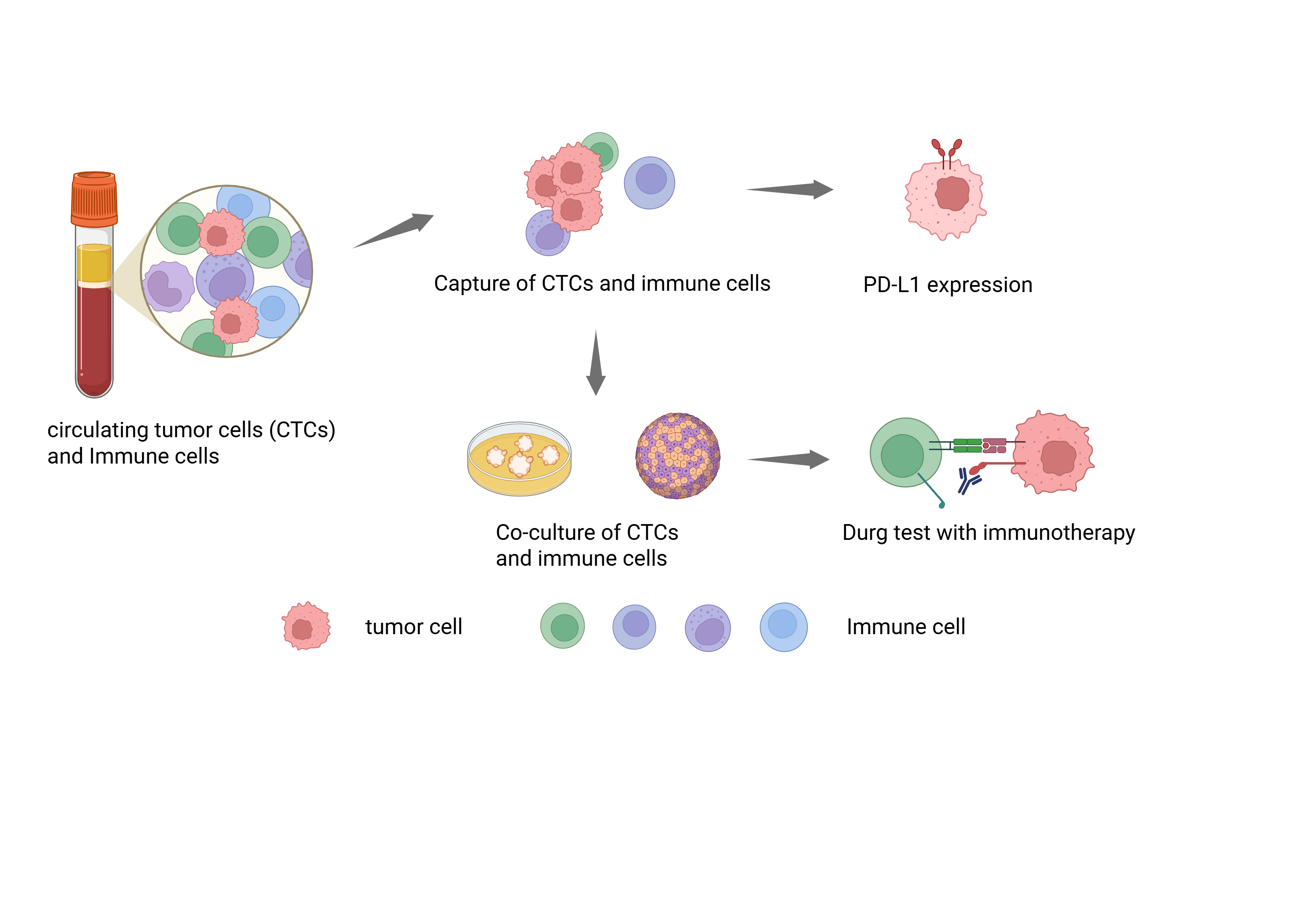


**Figure S5. A preliminary illustration demonstrating the co-culture of CTCs and immune cells for potential application in immunotherapy.** CTCs and immune cells isolated from peripheral blood of patients, which are co-cultured enabling visualization of immune–tumor cell interactions and subsequent response profiling to immunotherapy.

| **Table S1.** The CTCs cultures have been applied for drug screening in cell lines or patients with cancers | | | | |
| --- | --- | --- | --- | --- |
| **Year** | **Cancer type** | **Culture types** | **Sample number** | **reference** |
| 2014 | estrogen receptor-positive breast cancer | breast cancer CTC cell line | 6 | Science. 2014, 345(6193):216–220. |
| 2016 | hepatocellular carcinoma (HCC) | CTCs/ CTCs spheroid | 2 | Cancer Biology and Therapy 2016, 17:1177-1187 |
| 2017 | small-cell lung cancer (SCLC) | SCLC CTC cell lines | 9 | Transl Lung Cancer Res. 2017 6(4):397–408. |
| 2020 | prostate cancer | CTCs | 3 | Cancer Drug resist. 2020, 3(3):636-646. |
| 2022 | breast cancer | CTCs/ CTCs spheroid | 1 | iScience, 2022, 25(10):105081 |
| 2022 | pancreatic ductal adenocarcinoma | CTCs spheroid | 31 | Eur J Cancer, 2022, 166:208-218 |
| 2022 | lung cancer | CTCs cluster | 20 | Med Oncol. 2022, 40(1):1. |
| 2022 | colorectal cancer | CTCs spheroid | 6 | Cancer Res. 2022, 41(1):86. |
| 2023 | thymic malignancies | CTCs spheroid | 12 | Thorac cancer 2023, 14(25):2591-2600. |
| 2023 | Primary Intracranial Ependymomas | CTCs | 1 | Diagnostics 2023, 13(7):1232. |
| 2023 | HCC | HCC cell line and 20 HCC patients | 20 | Adv Sci (Weinh). 2023, 10(14):e2206812. |
| 2024 | gastrointestinal Cancers | CTCs spheroid | 9 | Anticancer research 2024, 44(8):3481-3491. |

**Table S3.** Primers used in this study

| **Gene** | **primer name** | **Sequence (5'->3')** | **Product length** | **Note** |
| --- | --- | --- | --- | --- |
| HER2/neu (ERBB2) NM_001005862.2 | HER2-F' | TCACAGATAAAACGGGGGCA | 145 | for SYBR green qPCR |
|  | HER2-R' | GGTGCACACTCSACTTTTGTGC |  |  |
| ESR1 (NM_000125.3, NM_001122740.1) | ESR1-F' | TATGTGTCCAGCCACCAACC | 119 | for SYBR green qPCR |
|  | ESR1-R' | GGTCTTTTCGTATCCCACCTT |  |  |
| PGR (NM_000926.4, NM_001202474.3) | PGR-F' | TCAACTACCTGAGGCCGGAT | 134 | for SYBR green qPCR |
|  | PGR-R' | GCTCSCCACAGGTAAGGACAC |  |  |
