# Supplementary material for: Application of CTC-derived spheroid for drug screening toward personalized treatment in patients with breast cancer
Source: Transl Oncol. 2025 Oct 27;63:102573. doi: 10.1016/j.tranon.2025.102573 (PMC12597000; doi:10.1016/j.tranon.2025.102573)
Supplement: Supplementary file 2 [file mmc2.docx]

**Table S4. The patients enrolled for drug test using CTCs spheroid culture**

| Patient number | Stage | Subtype | Drug history before drug test | Sensitive drug tested by CTC spheroid culture | Comparable used drugs after drug test | Clinical response | Duration of treatment (months) |
| --- | --- | --- | --- | --- | --- | --- | --- |
| 1 | IV | HR+/HER2+ | P+H+D | LT+5-FU | LT + X | PR | 15 |
| 2 | IV | HR+/HER2+ | P+H+D | H+D | P+H+D | PR | 9 |
| 3 | IV | TNBC | H+D+CDDP, C+E+5-FU, B+V+X | G | T + G | PR | 2 |
| 4 | IV | HR+/HER2- | L+PL | Nil | L + F | PD | 2 |
| 5 | IV | HR+/HER2+ | P+H+D, T-DM1 | LT + 5FU, T-DXd | T-DXd | PR | 3# |
| 6 | IV | HR+/HER2- | R+TA | E | C + E + 5-FU | PR | 2 |
| 7 | IV | HR+/HER2- | TA, L+GN, V+X+A, T+G. LP, Er, SG, T-DXd | G, 5-FU + EV, D + Cb, T+G | EV + X | PR | 12# |
| 8 | III | TNBC | N/A | D + CDDP | D + CDDP | PR | 3* |
| 9 | IV | HR+/HER2- | L + PL | Nil | EX + EV | SD | 3 |
| 10 | II | HR+/HER2- | E+C, D | Nil | TA | N/A | 49 |
| 11 | II | HR+/HER2+ | D+H+P | Nil | D+H+P | PR | 3* |
| 12 | IV | HR+/HER2+ | D+H+P | Nil | T-DM1 | PR | 33 |
| 13 | IV | HR-/HER2+ | D+H+P+Cb, E+C, T-DM1 | LT + X | LT + X | SD | 7 |

**Abbreviation:** HR: hormone receptor, HER2: human epidermal growth factor 2, 5-FU: 5-fluorouracil, A: Anastrozole, B: Bevacizumab, C: Cyclophosphamide, Cb: Carboplatin, CDDP: Cisplatin, D: Docetaxel, E: Epirubicin, Er: Eribulin, EV: Everolimus, EX: Exemestane, F: Fulvestrant, G: Gemcitabine, GN: Goserelin, H: Trastuzumab, L: Letrozole, LP: Lipo-doxorubicin, LT: Lapatinib, N: Neratinib, P: Pertuzumab;; PL: Palbociclib, R, Ribociclib, SG: Sacituzumab govitecan, T: Paclitaxel, T-DM1: Trastuzumab emtansine, T-DXd (DS-8201): Trastuzumab deruxtecan, TA: Tamoxifen, X: Capecitabine, CR: Complete response, PR: Partial response, SD: Stable disease, PD: Progressive disease, N/A: not applicable

**Footnote:** *Planned 4 cycles, #ongoing
